# Optically Guided Epidural Needle Placement Using 405-nm Wavelength for Accurate Puncture

**DOI:** 10.1038/s41598-018-38436-z

**Published:** 2019-02-07

**Authors:** Su-Man Lin, Cihun-Siyong Alex Gong, Tai-An Chiang, Mei-Yung Tsou, Chien-Kun Ting

**Affiliations:** 1 Department of Anesthesiology, Taipei Veterans General Hospital and National Yang-Ming University School of Medicine, Taipei, Taiwan Republic of China; 2 Department of Electrical Engineering, School of Electrical and Computer Engineering, College of Engineering, Chang Gung University, Taoyuan, 33302 Taiwan Republic of China; 3 Portable Energy System Group of Green Technology Research Center, Chang Gung University, Taoyuan, 33302 Taiwan Republic of China; 4 Department of Ophthalmology, Chang Gung Memorial Hospital, Linkou, Taoyuan, 33305 Taiwan Republic of China; 5 EDA Medical devices Technology Inc., 2F, No. 30, Kaya Road, 42881 Daya Dist., Taichung City, Taiwan Republic of China

## Abstract

Several approaches of locating the epidural space have been proposed. However, loss of Resistance method (LOR) remains the most common method for epidural anesthesia. Different optical signals were received from the ligamentum flavum and the epidural space allows operator to pinpoint position of the needle and determine whether the needle tip has entered the epidural space. Optical signals throughout the penetration process was recorded and position of needle tip was confirmed with a C-arm fluoroscopy. 60 lumbar punctures were performed in 20 *vivo* porcine models, and success rate of locating the epidural space with the optical auxiliary is calculated statistically. The data are expressed in mean ± SD. During all the lumber puncture processes, the strength of optical signals received decreased significantly while the needle tip penetrates the ligamentum flavum and entered the epidural space. The strength of optical signal received when needle tip was in the ligamentum flavum was 1.38 ± 0.57. The signal strength at epidural space was 0.46 ± 0.35. Strength of signal decreased by 67% when entered epidural space, and there is no significant differences in decrease of strength from data obtained from thevertebrae (lumbar segments)L2-L3, L3-L4, and L4-L5. Finally, we calculated with assistance of the proposed optical auxiliary, the success rate for guiding the needle tip to the epidural space using was as high as 87%. It is evidently believed that the optical auxiliary equipped is visualized to assist operators inserting needle accurately and efficiently into epidural space during epidural anesthesia operation.

## Introduction

Loss of resistance (LOR) generally used with air or saline is the most common method for epidural puncture operations^1–4^. However, statistics shows 10% of the epidural operations failed^5,6^, due to incorrect needle tip positions failing to achieve anesthesia effect^7,8^. It is suggested the success rate depends heavily on operators’ experiences, patients’ weights, medication histories, and epidural anatomy^9,10^. Moreover, it is observed LOR is typically time-consuming, difficult to familiarize and frequently accompanied with postoperative-syndrome^11,12^. As a result, it is critical to develop an accurate method capable of locating the epidural space during the epidural puncture.

Hanging drop technique also uses “decrease in pressure” concept to identify the epidural space, however it is more likely to lead to dural puncture as compared to LOR; therefore it is rarely used clinically^13,14^. Ultrasonography and fluoroscopy methods are another two approaches to assist operators locating needle tip during epidural puncture^15^. Ultrasound imaging technique presents the distance between the needle tip and the epidural space while also displays the spine column and its surrounding structures^16^. Ultrasonography demonstrates capability of improving accuracy and safety significantly for needle positioning, but is unable to differentiate the tissues from the epidural space due to its poor resolutions. Furthermore, it cannot be operated independently as it requires one person to operate an ultrasound probe and another person do execute epidural puncture^17^. Fluoroscopy technique also uses visual guidance to assist locating position of the needle tip^18^. Combination of Fluoroscopy and LOR not only improves the accuracy of needle location but also reduces the incidence of complications significantly^19^. However, fluoroscopy approach could not identify the forms of tissue around the needle tip, not to mention soft tissues like blood vessel and nerves^18,19^. Pressure wave method can detect the pressure change from ligamentum flavum to epidural space as the needle tip progresses towards epidural space and in turn converts to audio signals to guide the operator^20–22^. Bioelectrical impedance method is another location technique, which identify different tissues through their different electronic characteristics, such as muscles or lipids, and high impedance of epidural spaces^23^. However, the signals can easily be interfered by membrane potentials of the nerve^24^.

Ting *et al*. embedded optical fibers within a Tuohy needle in order to emit 650 nm or/and 532 nm laser beam (s) and to receive reflected optical signals from the tissues in porcine models^25^. Optical signals reflected by each of the tissue layers were analyzed to guide the needle tip to the epidural space or the ligamentum flavum^25^. Furthermore, the fiber-embedded needles is able to provide visible and near-infrared reflection spectra which indicates the percentages of blood and lipid contents in the analyzed tissues while the epidural puncture was executed with median and paramedian puncture method^25^. In this study we improved further the mechanical design of the fiber-embedded needles and replaced the 650 nm and 532 nm laser beams with 405 nm laser beam. A signal processing device was developed to convert the received optical signals to digital data and display them on screens, turning out the new design is capable of providing feedback to assist operators performing epidural block by accurately locating the epidural space for needle placement.

## Materials and Methods

### Study design

**Figure 1:**
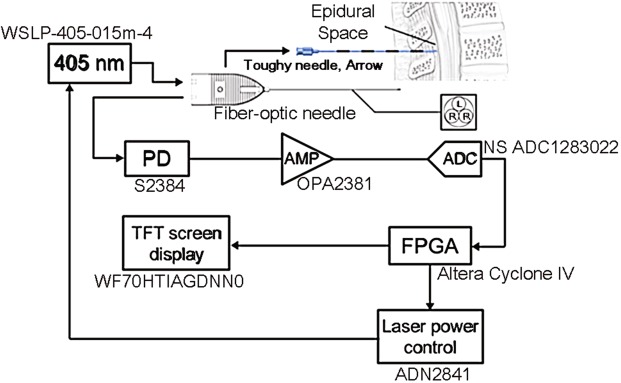
Optical locator function diagram. PD: Photo diode. AMP: Amplifier. ADC: Analog-to-digital converter. FPGA: Field-programmable gate array. L: Laser-end optical fiber (100 µm). R: Receiving-end optical fiber. Details of the used devices are shown. A 17-gauge Tuohy needle (Arrow, Teleflex Incorporated, Limerick, PA), was embedded with a 19-guage (diameter: 1.1 mm) metal tubes, and a 100-µm and two 200-µm optical fibers within each tube.

Ting *et al*. has verified that the reflective signals from ligamentum flavum and the epidural space have substantial differences in *invivo* porcine models during epidural puncture operation^25^. As 650 nm/532 nm laser beams were relected from the ligamentum flavum and epidural space, the signal strength of the reflected optical signal from ligamentum flavum was significantly higher than that of the epidural space^25^. We found that 405-nm laser beam is able to provide good reflective optical feedback in various tissues, especially the collagen of the ligamenta flavum as a contrast. Unlike near-infrared beams, the 405-nm laser beam is able to provide high-intensity optical feedback in fat tissues (a.k.a. dense connective tissue)^26^. As a result, in this study, we attempt to perform epidural puncture using 405-nm violet laser beams to guide epidural needle placement and to verify the ability of the 405-nm laser beam to identify different tissues. Two goals have been achieved in this study: 1. With aids of the optical auxiliary, an operator is able to successfully perform the epidural puncture by accurately placing the needle tip to the target regions; 2. Data on locator apparatus is able to provide clear warning indicating the timing an operator should stop proceeding with punctuation immediately when dural puncture could occur during epidural puncture, where data at that specific moment can be recorded and analyzed. The proposed optical location system and its associated function diagram can be seen from Fig. 1.

### *In Vivo* study

#### Porcine Model

30 LYD crossbred pigs with average weight distributions of 40 ± 5 kg, 50 ± 5 kg, and 60 ± 5 kg were used as *in vivo* porcine models. All pigs were fasted and cleaned 12 hours before the study. Tranquilizer including stresnil (3.5 mg/kg, Janssen pharmaceutica NV, Lot no. DBB2P00) and atropine (0.03–0.05 mg/kg, Taiwan biotech Co. Ltd, Lot no. 2HS3077) were injected into the muscles of the porcine models first, then anesthesia inducing agent Zoletil 50 (4–5 mg/kg, Panion& BF biotech Inc., Lot no 4900-1401) was injected 20–30 minutes later in order to induce the effects of the tranquilizers. The porcine models were fixed on the operation tables in their prone position and NIM EMG tubes were inserted. Medications (Isoflurane 0.5–2% in nitrous oxide: oxygen, 2:1 or oxygen only) were injected with a flow rate of 3 L/min to maintain anesthetic effect. A fiber embedded with metal needle was introduced into a Tuohy needle (Arrow, Teleflex Incorporated, Limerick, PA) to perform median puncture to the L2-L3, L3-L4, and L4-L5 lumbar vertebras of the porcine models. One insertion was conducted for each vertebra and the optical auxiliary was utilized at all times to guide needle tip placement. As the needle tip entered the fat tissue regions, the optical auxiliary exhibits high-intensity reflections with feedback data up to full-scale (255). As the needle pinpoints enter the supraspinous ligament, interspinous ligament, and ligamentum flavum regions, the optical auxiliary shows no recognizable difference when crossing those tissue boundaries. However, the optical reflections decayed abruptly, indicating the needle tip had entered a geometric void. This is the reason behind the abrupt decaying of optical signal. When it occurred, we assumed the needle tip had entered epidural space and stopped progression of needle insertion. We used C-arm fluoroscopy (OEC 7900 Flurostar GE Healthcare, UK) to verify the location of needle tip (N = 20). Furthermore, an experiment was designed to examine the scenario when the needle pinpoints reached or pierced through the dura mater. We observed once the needle tip had successfully entered the epidural spaces. Advancing further the needle resulted in increased reflection signal intensity. We first assumed that this was because the needle tip had reached with dural mater, followed by verification with C-arm fluoroscopy (N = 10). All porcine models were sacrificed immediately after the experiments. The experimental protocols (Protocol IACUC 2015-035) used in this work were approved by the Institutional Animal Care and Use Committee of Taipei Veterans General Hospital. The animal protocols and all the associated methods are in accordance with Council of Agriculture Executive Yuan Guideline for the Care and Use of Laboratory Animals, Taiwan, under GUIDE FOR THE CARE AND USE OF LABORATORY ANIMALS (Eighth Edition) updated by National Research Council (US) Committee.

#### Optical-guided Location Device

The optical-guided location auxiliary consists of two parts: an optical fiber embedded needle probe and an optical-to-digital-signal converter. A 17-gauge Tuohy needle (Arrow, Teleflex Incorporated, Limerick, PA), was embedded with a 19-guage (diameter: 1.1 mm) metal tubes, and a 100-µm and two 200-µm optical fibers within each tube. The needle tips were polished to provide smooth surfaces for optical functions. The 100-µm optical fibers transmit 405-µm laser beams and the 200-µm optical fibers transmit reflected light from the irradiated tissues. The reflected optical signals are feedback to the location device for real-time analysis. Also, a grip was added on end of the probe to improve operability and to avoid accidental and undesired needle displacement. The optical-to-digital-signal converter supplies power (5 mW) to the 405-µm laser diodes and receives reflected optical signals from the irradiated tissues, followed by converting the optical signals into digital data. Digital data go through digital amplifiers and then are sent to FPGA (Field Programmable Gate Array) for sampling and mean of the data was calculated. The amplified digital signals were quantified on scale of 0 to 255 and displayed on TFT screen. Purpose of the converter is to indicate the intensities of reflected optical signal from the various tissues, thereby allowing operators to accurately guide the needle tip into the epidural spaces with the help of change in optical signal when needle is entering epidural space from ligament flavum.

#### Statistical Analysis

The received reflection light intensities from the ligamentum flavums and epidural spaces were statistically analyzed. Photo diodes have been used to capture and convert the reflected optical signals into electric signals then information interceptors (ADVANTECH, USB-4704) will capture and standardize the data to relative strength. Data was retrieved seven times at lumbar vertebra L2-L3, eight times at lumbar vertebra L3-L4, and six times at lumbar vertebra L4-L5. The data collected at the ligamentum flavums and the epidural spaces were averaged and compared. Data from other lumbar vertebrates segments (L2-L3, L3-L4, L4-L5) were also compared and presented in mean ± SD displayed in a bar chart. Data were compared with t-test in order to test the significance of the differences. Finally, the data and success rate of identifying and reaching the epidural space during epidural puncture were displayed in charts.

## Results

**Figure 2:**
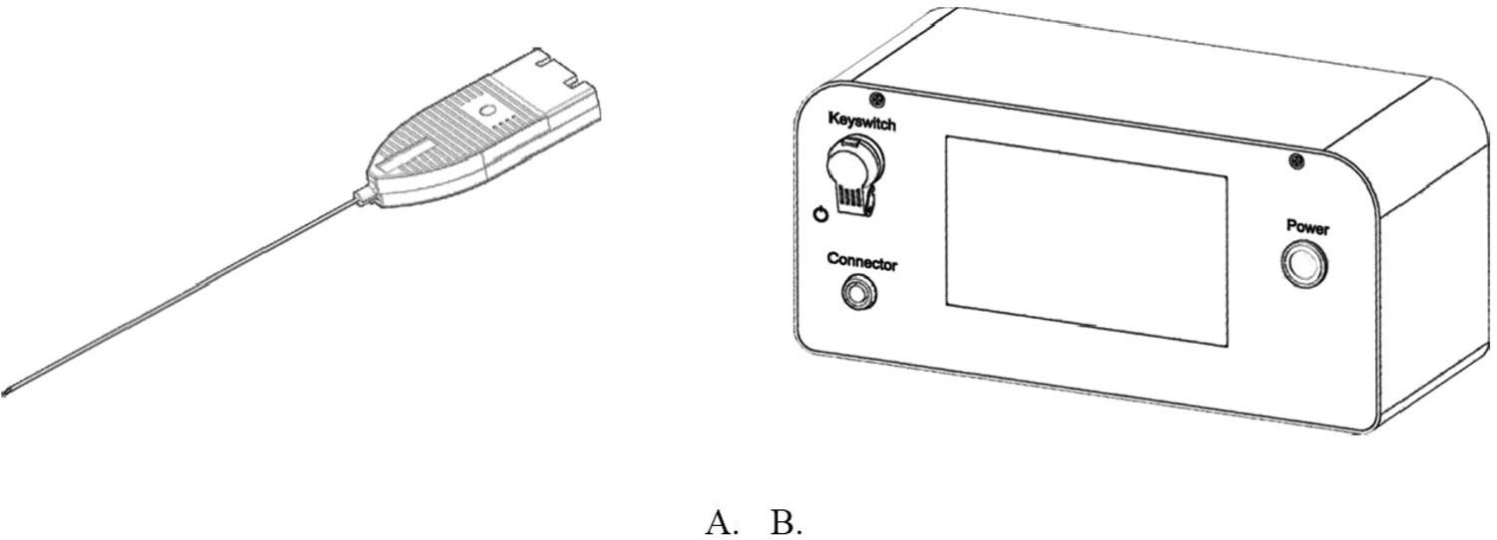
Optical-guide Location Device Appearance and Interface. (**A**) Epidural Space Location Needle: A probe embedded with one 100-µm and two 200-µm optical fibers. 200-µm fibers receive reflected light from the tissues irradiated by the 405-nm laser emitted through the 100-µm fiber inside the needle probe. (**B**) Epidural Space Locator apparatus: A power source supplies power to the laser diode emitting 405-nm laser beam, and converts the reflected optical signals to digital data and display data on a TFT screen.

**Figure 3:**
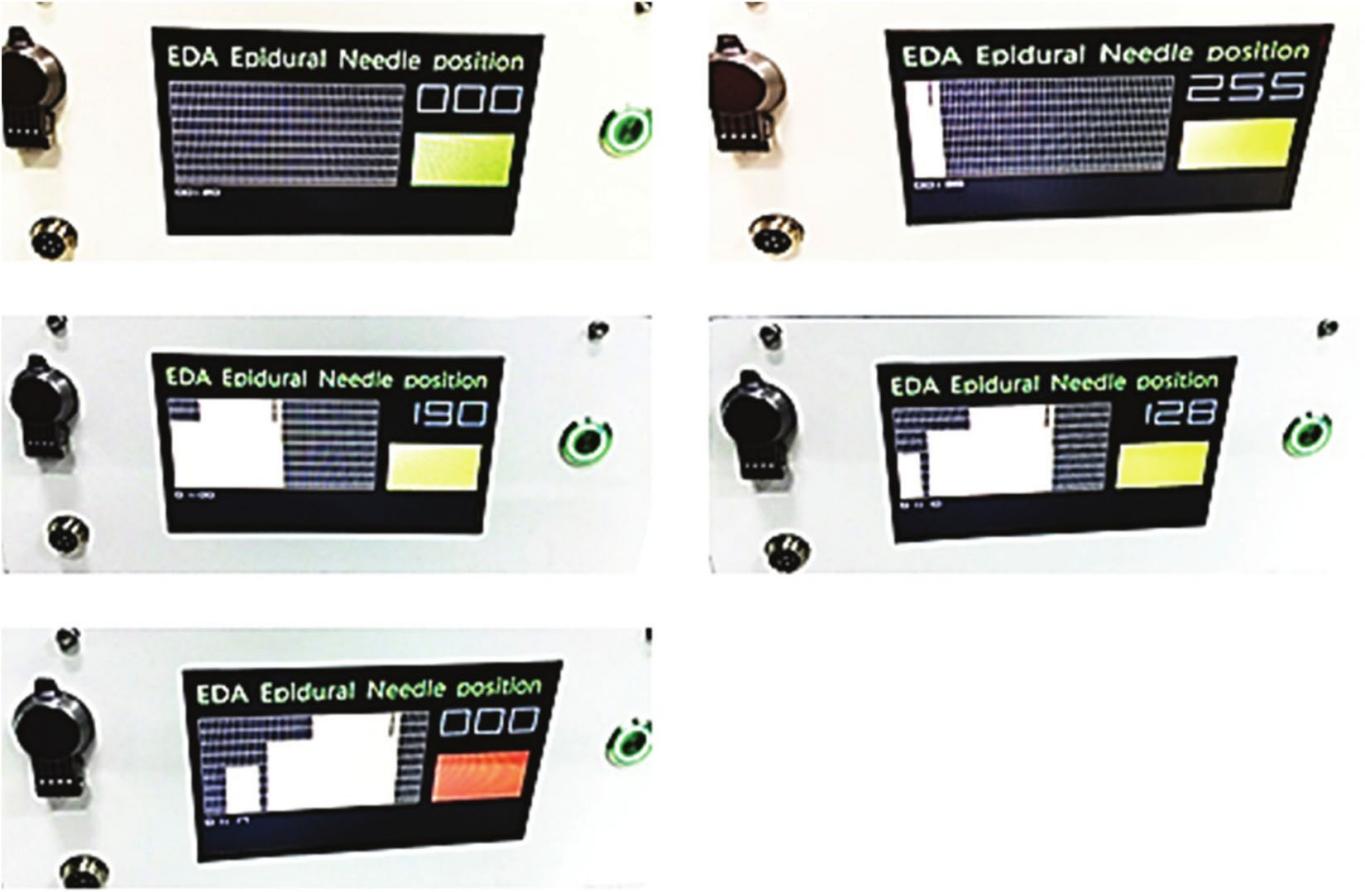
Optical-guided Location Device data displays. When reflection intensity is 0 meaning no reflected light received, in this case color bar will be green; When reflection intensity reaches 255 the color bar will turn yellow meaning needle tip has reached fat layer tissue; When reflection intensity decreases with advance of needle tip and drops to zero color bar will turn red bar indicating that the needle tip has reached the epidural space.

This research used a developed optical auxiliary to assist operators accurately guiding the needle tip into the epidural spaces by referring to the real-time data of the reflected optical signals from tissues. The optical signals are converted into digital data through a converter. The optical auxiliary consists of the epidural space location needle (Fig. 2A) embedded with optical fibers combined with the epidural locator apparatus. A laser diode at the needle tip was powered to emit 405-nm laser beams. The reflected light from various tissues were converted into digital data subsequently displayed on screens. These processes assist operators to determine the location of the needle tip (Fig. 2B), in which the digital data decay as the intensity of the reflected light (Fig. 3).

**Figure 4:**
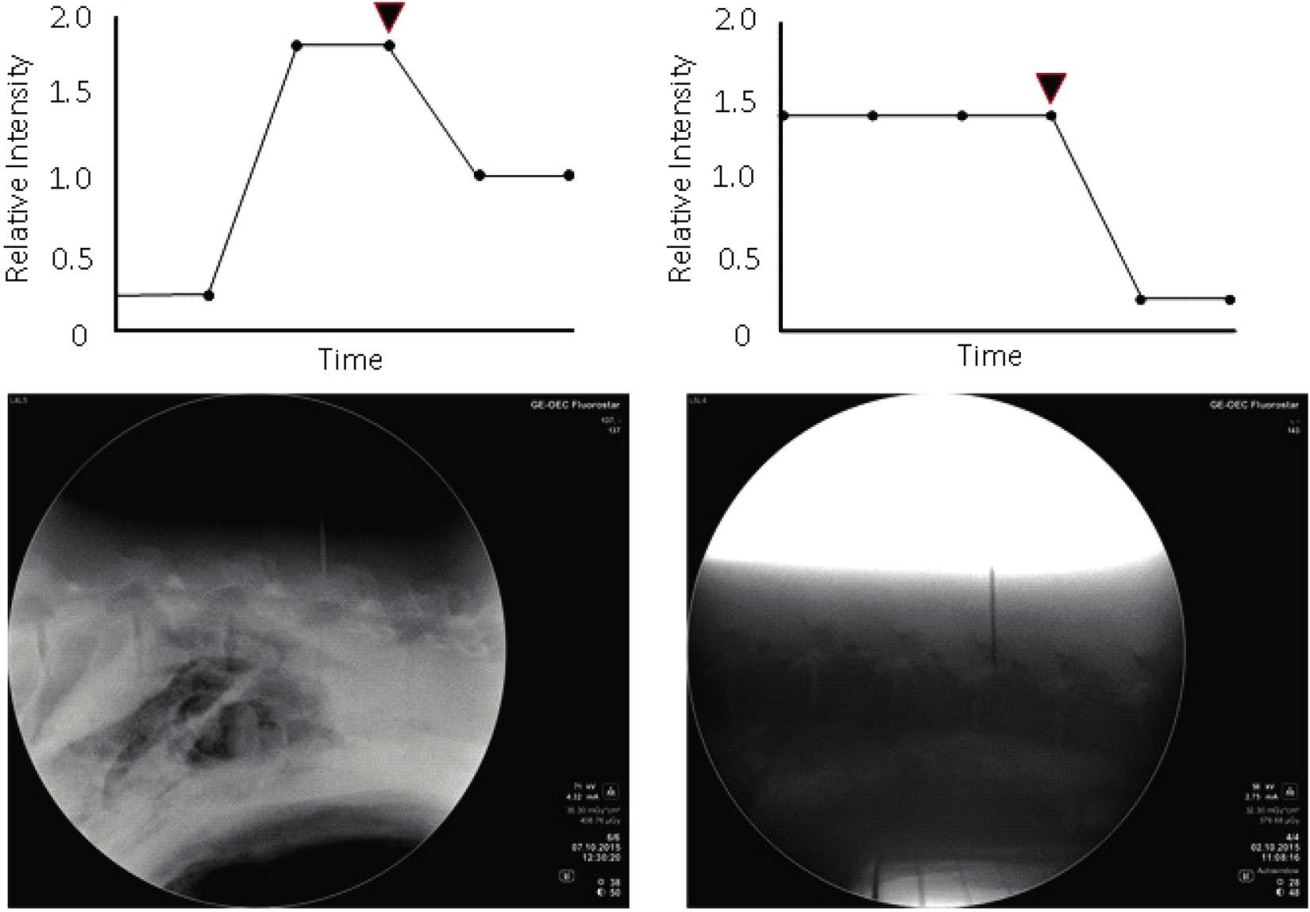
Data change during lumbar puncture using proposed optically guided location device for dural puncture operation. Black triangles indicate timing locating ligamentum flavum. The radiography confirms correctness of needle placement.

90 epidural punctures were performed on the L2-L3, L3-L4, and L4-L5 vertebrae of the 50.75 ± 9.92 kg porcine models in this research. 60 times of the executions were conducted until the needle tip entered the epidural spaces where data reading decreased to 0 from received optical signal. The accuracy of the needle tip location was verified by C-arm fluoroscopy. Figure 4 shows the selected examples showing variations of the reflected signals in 21 needles punctured into the vertebrae in eight porcine models. When median epidural puncture was performed, we found that the reflected signal decreased significantly as the needle tip entered the epidural spaces from the ligamentum flavum (P < 0.001). The reflection relative intensities decreased by 0.92 ± 0.22 from 1.38 ± 0.57 to 0.46 ± 0.35 as the needle tip entered the epidural space from the ligamentum flavum. The intensity of reflected optical signal decreased by 67% when needle tip reaches epidural spaces. We then analyzed the decrease in intensity of optical signals when needle tip was entering the epidural space at different vertebrae. The intensity decreases at vertebrae L2-L3, L3-L4, and L4-L5 were 0.81 ± 0.45, 0.98 ± 0.74, and 0.98 ± 0.62 respectively. No obvious deviations between groups were observed.

Finally, we statistically analyzed the success rate and real-time data during epidural puncture using optically guided locator apparatus and displayed data in Table 1. Furthermore, additional 30 epidural puncture were performed for verification. The entire procedures were monitored and also verified through the C-arm fluoroscopy process to examine whether the needle tip had penetrated the epidural space (Table 2). With the developed optically assisted auxiliary, the operators succeeded 52 times out of 60 times in accurately placing the needle into the epidural spaces. Success rate of accurate positioning was 87% (89.7% if excluding two cases of human operation error). Once the needle tip had reached the epidural space, further advance of needle will result in alarming signal from Locator Apparatus will be triggered and reading will increase again when needle tip had reached or punctured through the dura mater (Table 3).

**Table 1:** Reflection light intensities when the Optical-Guided Location Device guides the needle tip to epidural space.

Animal I.D.	Body weight (kg)	Number of lumbar punctures	Reflect optical signal (value)		
			L2-L3	L3-14	L4-L5
127	60.2	3	0*	0	0
130	65.0	3	0*	0	0
131	62.5	3	0	0	0
132	61.5	3	0	0	0
133	60.1	3	0	0	0
155	58.0	3	0	0	0
140	55.0	3	0	0	0
137	55.5	3	0	0	0
139	55.0	3	0	0	0
138	54.2	3	0	0	0
153	52.0	3	0*	0	0
141	50.0	3	0*	0	0
142	40.0	3	0	0	0*
146	42.0	3	0*	0	0
144	40.0	3	0*	0	0*
149	40.5	3	0	0	0
143	38.0	3	0	0	0
145	37.5	3	0	0	0
151	38.0	3	0	0	0
154	49.0	3	0	0	0

*indicates failure of needle tip reaching epidural spaces or erroneous epidural puncture.

**Table 2:** Data displayed on Optical-Guided Location Device for reflected optical signal when the needle tip reached the dura mater.

Animal I.D.	Body weight (kg)	Number of lumbar punctures	Reflect optical signal close to 0			Reflect optical signal (value)		
			L2-L3	L2-L3	L3-14	L4-L5	L3-14	L4-L5
156	63.5	3	Yes	Yes	Yes	128	128	128*
159	61.0	3	Yes	Yes	Yes	128	128	128
157	60.0	3	Yes	Yes	Yes	128	190	128*
158	60.5	3	Yes	Yes	Yes	128	128	128
129	54.5	3	Yes	Yes	Yes	128	128*	128
128	53.0	3	Yes	Yes	Yes	128	128	128
136	50.0	3	Yes	Yes	Yes	128	128	128
148	36.0	3	Yes	Yes	Yes	128*	128	107
147	35.0	3	Yes	Yes	Yes	128	128	128
150	35.0	3	Yes	Yes	Yes	128	128	128*

^#^Reflected optical signal during further advancing of needle tip after reading drops to 0 when epidural space was reached.

*Reflected optical signal where no needle tip entered epidural spaces or needle tip was inserted at the wrong vertebrae.

**Table 3:** The optical locator performs in a lumbar puncture arrival success rate of epidural/spinal dura mater.

Placement	Number of animals	Number of lumbar puncture	Overall successful lumbar puncture rate^†^	Successful lumbar puncture rate
To epidural space	20	60	52/60 (87%)	52/58 (89.7%)^a^
To duty mater	10	30	25/30 (83.3%)	25/25 (100%)^b^

^†^Number of successful lumbar puncture/total number of lumbar puncture.

^a^Two failed lumbar punctures due to human error from operator were excluded.

^b^Five failed lumbar punctures due to human error from operator were excluded.

## Discussion

Similar to the studies conducted by Ting *et al*.^25^, this research also implements optical technologies in guiding the needle tip to epidural space with precision, except that 532-nm or 650-nm laser beams have been replaced with 405-nm laser beams, which were experimentally verified to have more significant reflection characteristics as the needle tip entered the epidural space in *in vitro* porcine model study. Also, the operability of the optical-fiber embedded needle probe has been improved and combined with subsequent image processing IC devices to display data on LCD screens instead of traditional oscilloscopes. During this *in vitro* porcine model study, the components for the improved needle probe have been modulated based upon the 405 nm laser beam specifications. We observed a specific optical phenomena occurred when needle tip entered the epidural space of lumbar vertebra. When entering, the optical signal was characterized by staged reduction of light reflections from tissues^27^, identical to the dramatic resistance decay during epidural puncture with LOR technique. Given that epidural space is not an open anatomical space, it is usually laying on the surface of the vertebral canal, therefore reasonably comprehensive that the reflection light beams will scatter and leak around after the needle tip had entered the epidural space, hence less amount of light reflected. This results in the decay of the reflected optical signal.

This research first observed the effectiveness of the 650-nm laser beam of the Ting’s studies as compared to the 532-nm laser beam during the epidural puncture, followed by the developed optical-assisted auxiliary having improved the system by replacing 532-nm and 650-nm laser beams with 405-nm laser beam. The idea is intended to obtain high contrast images during epidural punctures, and also have modulated the system components to improve location accuracies. All improvements have been verified through epidural puncture experiments practiced on porcine models. Acquired signals were recorded and analyzed statistically to calculate probability, which indicates success rates of needle tip entering epidural space with optically assisted auxiliary. During epidural punctures intensity of reflected optical signal increased rapidly as needle tip enter subcutaneous fat layers then decreased rapidly as needle tip entered muscle layers. Once the needle tip pierces into interspinous ligaments, reflection intensity started to increase again and maintained at the same magnitude until ligamentum flavum was reached. Once the needle tip entered epidural space from ligamentum flavum, reflection intensities drop abruptly, which implies the pinpoints have successfully reached the epidural space.

Study conducted by Rathmell *et al*.^28^ also uses probes with embedded optical fibers to study optical reflective spectrum of interspinous ligaments, muscles, ligamentum flavum, epidural fat, and cerebrospinal fluid^28^. It was observed that the epidural fat can absorb few light beams, and most optical energy are reflected back to the needle tip which provides high intensity feedback. Analyzing reflection optical spectra from muscles shows high blood fractions since muscles belongs to skeletal muscle. Two types of hemoglobin content are found in compose blood: one is Deoxygenated hemoglobin (Hb) and the other is Oxyhemoglobin (HbO2). Deoxygenated hemoglobin (Hb) has primary absorption peak at 420 nm and secondary at 580 nm. Oxyhemoglobin (HbO2) has primary absorption peak at 410 nm, and secondary at 550–600 nm. Hence, large amount of optical energy is absorbed by Deoxygenated hemoglobin (Hb) and Oxyhemoglobin(HbO2) in blood as 405-nm laser beams enters muscle tissues. As a result, the reflected optical signal acquired at the needle tip decreases as expected. Interspinous ligament and ligamentum flavum are both categorized as connective tissue, and the blood and fat compositions are approximately the same. The reflection intensities show no obvious differences as the needle pinpoints passing through the two tissues. In anatomy, epidural spaces are not an open anatomical spaces but narrow gaps between Dura Mater and Vertebral Periosteum. Epidural spaces simply appear during anesthesia operations when Dura Mater and Vertebral Periosteum are intentionally pried apart. Hence, emitted laser beams scatter and leak through the gaps reflecting minimal optical signal through optical fibers. However, epidural space is not a vacant space, it is surrounded by lymphatic nodes, spinal nerve root, loose connective tissue, fatty tissue, small arteries, and extensive plexus of veins. In Rathmell’s study^28^, reflected optical signal from epidural fat during epidural puncture was used to locate the epidural space. Both Rathmell’s and our studies used optical method to locate epidural space but with different approaches. We consider that although there is rich fat content at epidural space and the distributions are predictable along the spinal canal, there are still cases where no detection was received even though the needle tip has entered the epidural space.

**Figure 5:**
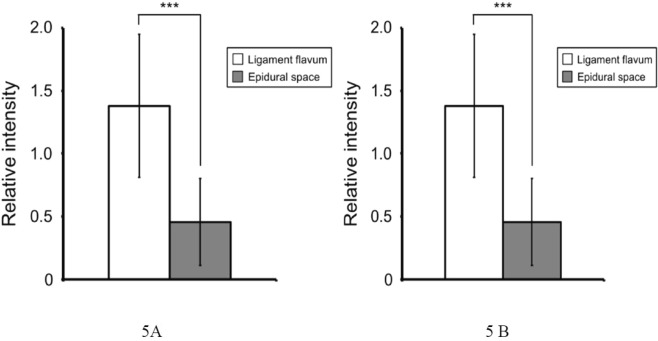
Signal change while the needle tip is entering epidural space from ligamentum flavum. (**A**) Reflected light intensities when needle tip penetrates the ligamentum flavum and enters the epidural space. (**B**) Decrease of reflection light intensities at different vertebrae while needle enters epidural space from ligamentum flavum.

Although there are several advantages that optical-assisted techniques can offer, reflection intensities could be affected by several factors within the tissue regions. For example, hemoglobin is one among them. We think blood could be responsible for the decrease of the optical signal in muscle. During epidural puncture, we detected interference when blood flows to the tissue around the needle tip when needle is inserted. There are other factors in tissue that could interfere with optical readings. For example, myoglobin in muscles and carotenes in epidural fat could also affect reflection intensities. Also, the fact that lengths of the needle tip surfaces or angle of the insertion could result in two receiving optical fibers are located in different tissues at that moment which would certainly affect the optical readings. We found that decrease in reflection intensities occur both when needle tip is located at epidural spaces as well as in muscles. Since 405-nm laser beam is close to the peak optical absorption spectra for Oxyhemoglobinand Deoxygenated hemoglobin, the reflection intensities are certainly weaker in tissues that contain more blood. Since experiments have not been conducted to identify optical properties for various tissues, we are unable to determine what the corresponding optical readings for different tissues are. However, it has been verified that the developed auxiliary system does assist locating epidural spaces on lumbar vertebrae, and reflection signals are basically the same from the segments exterior to the epidural spaces, as shown in Fig. 5B. However, experiments for epidural space location in other segments of the spine have not been conducted. It is awaiting for further verification in future research that the developed auxiliary is able to achieve the same goal in cervical vertebrae where the thinnest ligamentum flavum was found and Thoracic Vertebrae where the thickest ligamentum flavum was found within the whole porcine models.

The studies accomplished implementing 532-nm/650-nm and 405-nm laser light beams for locating epidural space, based on the dramatic changes in reflection intensity as needle tip enters epidural space from ligamentum flavum. Implementing 405-nm laser light beams actually demonstrates dramatic reflection differences, and edges the other two types of laser beams (532 nm/650 nm) due to its moderate reflections which is better for tissue differentiation. However, exercising the 405-nm laser also reveals reduction of reflection intensities as the needle tips are located in tissues with more blood contents. Nevertheless, in^24^, it has been demonstrated that their data provide evidence that as wavelengths decrease into the UVA, the dominant tissue chromophore shifts from hemoglobin to structural proteins such as collagen. It happened to be essential and important building component of the dense connective tissue which forms the ligamentum flavum. Furthermore, this research utilizes the optical energy absorption changes to differentiate ligamentum flavum and epidural fat, and therefore locates the epidural spaces. It is assumed that the ratios of blood and fat fractions are fixed for each type of tissue, and reflections of a particular laser beam from various tissues have a fixed pattern. Epidural fat is rich in exterior regions of the dura mater, thereupon dramatic decrease in reflection intensity is anticipated as the needle pinpoints entering epidural spaces from ligamentum flavum. However, fat and blood fractions of the same tissue may vary between individuals, and epidural fat could also have different distributions. Also, blood could follow needle insertion trace alters, flow into tissues, changes tissue blood contents, and subsequently affects tissue reflection characteristics, which may in turn result in dural puncture. As a result, there are still pros and cons for different approaches of optically assisted technologies, and eliminating experimental errors for different individuals is important for current researches.

The need of using two receiving-end optical fibers was to maximize the receiving sensitivity. The stylet has been custom made so that its size and shape are identical to the original one. The fibers at the tip of the needle have been glued together. They were shaped as bevel to fit the needle. Because the length of the fiber cable is only about 180 cm, the attenuation for the 405-nm wavelength can be neglected. It has been found that although there is no data of refractive index for the hardened glue available at present, this didn’t affect the results of our findings. The light does refract into the cladding layer and hit the cladding-tissue boundary. That is because that the *ex-vivo* porcine tissues have been reported of having refractive indexes higher than 1.27 so that the light traveling track in the fiber core can allow it to be transmitted into the tissues through the boundary. As a result, although the fiber-optical needle and the Touhy needle are misaligned, its influence to the guidance of the epidural space can be neglected.

In conclusion, this is the first research on epidural space location using an optically assisted auxiliary with 405-nm laser. The optical location system presented by Ting *et al*. has been redesigned to further demonstrate and exploit the optical characteristics differences between ligamentum flavum and epidural space. A real-time optical signal receiving system has been developed to assist operators to accurately perform epidural anesthesia while avoiding dural puncture. The optically assisted auxiliary is used in 60 epidural punctures in *in vivo* porcine models, and the success rate of accurately guiding needle tip to epidural space was 87%. As a result, it has been verified that 405-nm laser beams have high tissue differentiation ability for epidural puncture operations. Most importantly, the developed auxiliary is able to quantify tissue reflection intensities and help operators efficiently guide the needle tip quickly and precisely entering the epidural space. Last but not least, the anesthesia operations using the developed auxiliary are similar to the traditional procedures as compared to other technologies^29–38^.
